# Estrogen Regulation of Anti-Apoptotic Bcl-2 Family Member Mcl-1 Expression in Breast Cancer Cells

**DOI:** 10.1371/journal.pone.0100364

**Published:** 2014-06-27

**Authors:** Jennifer L. Schacter, Elizabeth S. Henson, Spencer B. Gibson

**Affiliations:** 1 Department of Biochemistry and Medical Genetics, University of Manitoba, Winnipeg, Manitoba, Canada; 2 Manitoba Institute of Cell Biology, CancerCare Manitoba, Winnipeg, Manitoba, Canada; University of South Alabama, United States of America

## Abstract

Estrogen is implicated as an important factor in stimulating breast cancer cell proliferation, and presence of estrogen receptor (ER) is an indication of a good prognosis in breast cancer patients. Mcl-1 is an anti-apoptotic Bcl-2 family member that is often over expressed in breast tumors, correlating with poor survival. In breast cancer, it was been previously shown that epidermal growth factor receptors up-regulate Mcl-1 but the role of estrogen in increasing Mcl-1 expression was unknown. In ERα positive cell lines MCF-7 and ZR-75, estrogen treatment increased Mcl-1 expression at both the protein and mRNA level. In two ERα negative cell lines, SK-BR-3 and MDA-MB-231, estrogen failed to increase in Mcl-1 protein expression. We found that ERα antagonists decreased estrogen mediated Mcl-1 expression at both the protein and mRNA level. Upon knockdown of ERα, Mcl-1 mRNA expression after estrogen treatment was also decreased. We also found that ERα binds to the Mcl-1 promoter at a region upstream of the translation start site containing a half ERE site. Streptavidin-pull down assay showed that both ERα and transcription factor Sp1 bind to this region. These results suggest that estrogen is involved in regulating Mcl-1 expression specifically through a mechanism involving ERα.

## Introduction

Estrogen has been implicated as an important factor in stimulating breast cancer proliferation and cell survival [1]. Presence of estrogen receptor alpha (ERα) is an indication of a good prognosis and ERα positive patients are often treated with hormonal therapy [1]. However, resistance to hormonal therapy occurs, creating a need for superior targeted breast cancer therapies [1].

Currently, ERα positive breast cancer patients are treated with anti-estrogen hormonal therapies such as the drugs Tamoxifen [2]. Tamoxifen antagonizes ERα by binding and inhibiting estrogen-induced activation of transcription [2]. This inhibition occurs through the blockage of activation function (AF) 2 sites on ERα. This leaves AF1 sites unaffected creating a potential mechanism for drug resistance [2]. While pure-estrogen antagonists, such as Fulvestrant (ICI-182,780) have been developed, alternative mechanisms of drug resistance can occur such as through growth factor signaling [2]. Understanding how estrogen contributes to drug resistance will be important in developing new strategies to treat ER α positive breast cancer.

Myeloid cell leukemia-1 (Mcl-1) is an anti-apoptotic protein that may have an important role in regulating drug resistance [3]. Mcl-1 is an anti-apoptotic Bcl-2 family member that is often over-expressed in breast tumors and correlates with poor survival in breast cancer patients [4]. Previous studies have shown that the Mcl-1 gene is located on chromosome 1q21 and is frequently amplified in many cancers including breast tumors [5]. Beroukhim et al. (2010) found that *Mcl-1* is amplified in approximately 11% of all cancers, with an amplification of approximately 4% in breast cancer.

Previous literature has demonstrated that Mcl-1 is a downstream target of epidermal growth factor (EGF) in many different types of cancer, including breast cancer [6], [7]. In addition, EGF mediated signaling cascades, such as the MAPK pathway, have been implicated in regulating Mcl-1 expression [6], [8]–[10]. These signaling cascades result in the up-regulation of transcription factors that may regulate Mcl-1 expression, such as Elk-1 and Stat-3 [6], [11]–[13]. EGF-mediated activation of NFκB has also been shown to up-regulate Mcl-1 expression [7]. Overall, this suggests that targeting Mcl-1 may provide a mechanism for overcoming drug resistance in breast cancer patients [14].

Currently, the role of estrogen in regulating Mcl-1 expression remains unclear. Previous literature demonstrates that estrogen may be involved in regulating the expression of Bcl-2 family members such as anti-apoptotic protein Bcl-2 [15]. Herein, we determined that estrogen receptor activation is involved in up-regulating Mcl-1 expression by binding to a specific estrogen response element (ERE) site in complex with Sp1 transcription factor within the Mcl-1 promoter.

## Materials and Methods

### Tissue Culture

Human breast cancer cell lines MCF-7, SK-BR-3 and ZR-75 were obtained from the American Type Culture Collection (ATCC, Manassas, VA, USA) in 2010. Human breast cancer cell line MDA-MB-231 was obtained from Dr. Leigh Murphy (University of Manitoba) in 2012. MCF-7, SK-BR-3 and MDA-MB-231 cells were grown in Dulbecco's modified essential medium (DMEM) buffer (Thermo Fisher Scientific, Waltham, MA, USA) supplemented with 10% fetal bovine serum and 100 units/mL penicillin and 100 µg/mL streptomycin. ZR-75 cells were grown in Roswell Park Memorial Institute medium (RPMI) buffer (Thermo Fisher Scientific, Waltham, MA, USA) supplemented with 10% fetal bovine serum and 100 units/mL penicillin and 100 µg/mL streptomycin. All cell lines were grown in a 37°C incubator with 5% CO_2_.

### Treatment of Cell Lines

All cells were grown in phenol red free white media for 5 days prior to treatment with estrogen. MCF-7, SK-BR-3 and MDA-MB-231 cells were grown in phenol red free Dulbecco's modified essential medium (DMEM) buffer (Thermo Fisher Scientific, Waltham, MA, USA) supplemented with 5% charcoal-stripped fetal bovine serum and 100 units/mL penicillin and 100 µg/mL streptomycin. ZR-75 cells were grown in phenol red free Roswell Park Memorial Institute medium (RPMI) buffer (Thermo Fisher Scientific, Waltham, MA, USA) supplemented with 5% charcoal-stripped FBS and 100 units/mL penicillin and 100 µg/mL streptomycin. In transfection experiments, cells were plated to ∼60–70% confluency. For chromatin immunoprecipitation and streptavidin pull-down assay, cells were plated to ∼80–90% confluency. For all experiments, β-Estradiol (Sigma-Aldrich, Oakville, ON, Canada) was made up in ethanol to a stock concentration of 10 mM and stored at −20°C. Tamoxifen (Sigma-Aldrich, Oakville, ON, Canada) was made up in ethanol to a stock concentration of 2 mM and stored at −20°C. Fulvestrant (ICI-182,780) was obtained from Sigma-Aldrich (Oakville, ON, Canada) and was made up in dimethyl sulfoxide to a stock concentration of 500 mM and stored at −20°C.

### Protein Lysate Preparation and Western Blotting

Whole cell protein lysates were prepared from cell lines using Nonidet-P40 (NP-40) buffer (20 mM Tris HCl pH 8, 137 mM NaCl, 10% glycerol, 1% nonidet P-40, 2 mM EDTA) supplemented with protease and phosphatase inhibitors (Roche Diagnostics, Mannheim, Germany). To perform western blot, equal amounts of protein were resolved using SDS/polyacrylamide gel electrophoresis. Afterwards, proteins were transferred onto a polyvinyl difluoride membrane (GE Healthcare Limited, Amersham Place, Little Chalfont, Buckinghamshire) and blocked in Tris buffered saline containing 0.1% Tween-20 and 5% skim milk powder. Membranes were incubated with primary antibody overnight at 4°C and then washed with Tris-buffered saline-Tween 20. Afterwards, membranes were incubated with secondary antibody conjugated with horseradish peroxidase for 1–2 hours at room temperature. After incubation, proteins were visualized onto autoradiography film using enhanced chemiluminescence (Thermo Fisher Scientific, Waltham, MA, USA).

### RNA Isolation and Quantitative Real-Time PCR

Total RNA was isolated using the Qiagen RNeasy Plus mini kit (Qiagen, Toronto, Ontario, Canada) according to the manufacturer's protocol. To perform qPCR, 100 ng RNA template was used in combination with iTaq Universal SYBR Green Supermix (Bio Rad, Mississauga, Ontario, Canada).The cycling and data collection were performed on a Bio Rad CFX Real-Time Detection System (Bio Rad, Mississauga, Ontario, Canada) using the supplied software. The following primers specific for Mcl-1 mRNA were used: forward: 5′-GCCAAGGACACAAAGCCAAT-3′; reverse: 5′-AACTCCACAAACCCATCCCA-3′. Two different housekeeping genes were used to standardize the results. Cyclophilin, using the following primers: forward: 5′-GCTGCGTTCATTCCTTTG-3′; reverse: 5′-CTCCTGGGTCTCTGCTTTG-5′ or, TATA binding protein (TBP) using the BioRad PrimePCR SYBR Green Assay pre-designed primer pair (Bio Rad, Mississauga, Ontario, Canada). The qPCR reaction was run as follows: 50°C for 10 minutes, 95°C for 5 minutes and then 40 cycles of 95°C for 10 seconds and 55°C for 30 seconds. In order to validate primer specificity, samples were run out on an agarose gel following qPCR reaction.

### siRNA Transfection

To perform siRNA transfection, GenePORTER-2 Transfection Reagent (Genlantis, San Diego, CA, USA) was used according to the manufacturer's protocol. A total of 2 µg of 3-pooled siRNA was transfected per sample using prevalidated siRNA duplexes that were obtained from Origene (ESR1 (ID 2099) Trilencer-27 Human siRNA). The following ERα siRNAs were used: 5′-ACCUUGCAGAUAUGUUUAACC AAGC-3′ (SR301461A), 5′-ACACCAUAGUAAUGUCUAAUAUUCA-3′ (SR301461B), and 5′-GGCAAAUAGAGUCAUACAGUAGCTC (SR301461C). Cells were transfected with either siRNA against ERα or control siRNA. Cells were treated with 10 nM estrogen overnight and total RNA was isolated 48 hours post transfection. Total RNA was isolated and quantitative real-time PCR was performed as previously described. In order to validate knockdown, western blot analysis was performed using antibodies against ERα and β-actin as a loading control.

### Promoter analysis

The full mcl-1 sequence including promoter, mRNA, 5′UTR, CDS and 3′UTR (locus AF147742, 6502 bp in total) was analyzed using AliBaba 2.1 with 80% stringency. Results were confirmed using Transfac Matrix Table, Rel.3.3 and PROMO v3.

### Chromatin Immunoprecipitation

ChIP was performed following estrogen stimulation using the EZ-ChIP Chromatin Immunoprecipitation kit (Millipore, Billerica, MA, USA) according to the manufacturer's instructions. DNA was isolated using EZ-ChIP Chromatin Immunoprecipitation kit Spin Columns and Bind Reagents A, B and C (Millipore, Billerica, MA, USA). DNA concentration was determined using PicoGreen dsDNA quantification assay (Invitrogen, Burlington, ON, Canada). RT-PCR and qPCR were performed with 0.1 ng ChIP DNA using primers specific to the Mcl-1 promoter: forward site 1: 5′-GCACCCGCCACCATCCCCAGCTAATTTTTCGTATTTTTTTT-3′; reverse site 1: 5′-GCCATTGCAACTGGCCCTGTTTGTTAGGAAACAAGTCTTGG-3′; forward site 2: 5′-CTTTACCACCTGATAAAATTTTACTTTATAAAGCATGAGAG-3′; reverse site 2: 5′-GTTTCTCTATTTAAGGAATGTCTAATCTTGTACAGCAACTA-3′. The fold enrichment values were calculated using the cT value of each ChIP sample compared to the cT value of 0.1 ng Input DNA.

### Steptavidin Pull-Down Assay

To assess ERα and transcription factor binding, HPLC purified biotin-labeled probes specific to the Mcl-1 promoter were ordered from Integrated DNA Technologies (Integrated DNA Technologies, Coralville, IA, USA). A total of 3 site-specific biotin-labeled probes, as well as a scrambled probe and 3 unlabeled probes were used. The probe sequences were as follows: forward site 1: 5′-CCAGGATGGTCTTGA TCTCCTGACCTCGTGATCTGCCCGCCTCAGCCTCC-3′; reverse site 1: 5′-GGAGG CTGAGGCGGGCAGATCACGAGGTCAGGAGATCAA GACCATCCTGG-3′; forward site 2: 5′-GGCGCACTCTCAGCTCACCGCAACCTCCGCCTCCCAGGTTCA AGCGATTC-3′, reverse site 2: 5′-GAATCGCTTGAACCTGGGAGGCGGAGGTTGC GTGAGCTGAGAGTGCGCC-3′; forward site 3: 5′-TTTCTCCATGTTGGTTGGGCT GGTCTCAAACTCCTGACCTCAGATGATTC-3′; reverse site 3: 5′-GAATCATCTG AGGTCAGGAGTTTGAGACCAGCCCAACCAACATGGAGA AA-3′. Cells were treated with estrogen (10 nM) for 24 hours followed by nuclear extraction. The lysates were pre-cleared with 50 µL streptavidin agarose beads (Life Technologies, Eugene, OR, USA) for 30 minutes at 4°C with rotation. Next, a binding reaction was prepared, including: 500 µg nuclear extract, 50 ng/µL Poly dI-dC (Sigma-Aldrich, Oakville, ON, Canada), 1/5 volume 5X Binding Buffer (50 mM Tris pH 7.5, 250 mM KCl, 5 mM DTT), and 100 nM biotin labeled probe. The binding reaction was incubated for 30 minutes at room temperature. Afterwards, 50 µL of streptavidin-agarose beads were added and the samples were incubated for 30 minutes. The beads were resuspended in 50 µL of 2X SDS Loading dye and the samples were boiled for 5 minutes. Finally, SDS/polyacrylamide gel electrophoresis and western blotting were performed. Antibodies specific to ERα, ERβ, Sp1 and Sp3 were used. Cold-competition was performed was adding an excess of unlabeled probe 15 minutes before the addition of the biotin labeled Mcl-1 probe.

### Statistical analysis

Graphs were created and statistics were performed using GraphPad Prism4 software. Unless otherwise noted, a paired or unpaired two-tailed t test was performed according to the nature of data. Statistical significance noted in figures as *. Densitometry calculated using ImageJ.

## Results

### Estrogen treatment increases Mcl-1 expression in ERα+ breast cancer cell lines

**Figure 1:**
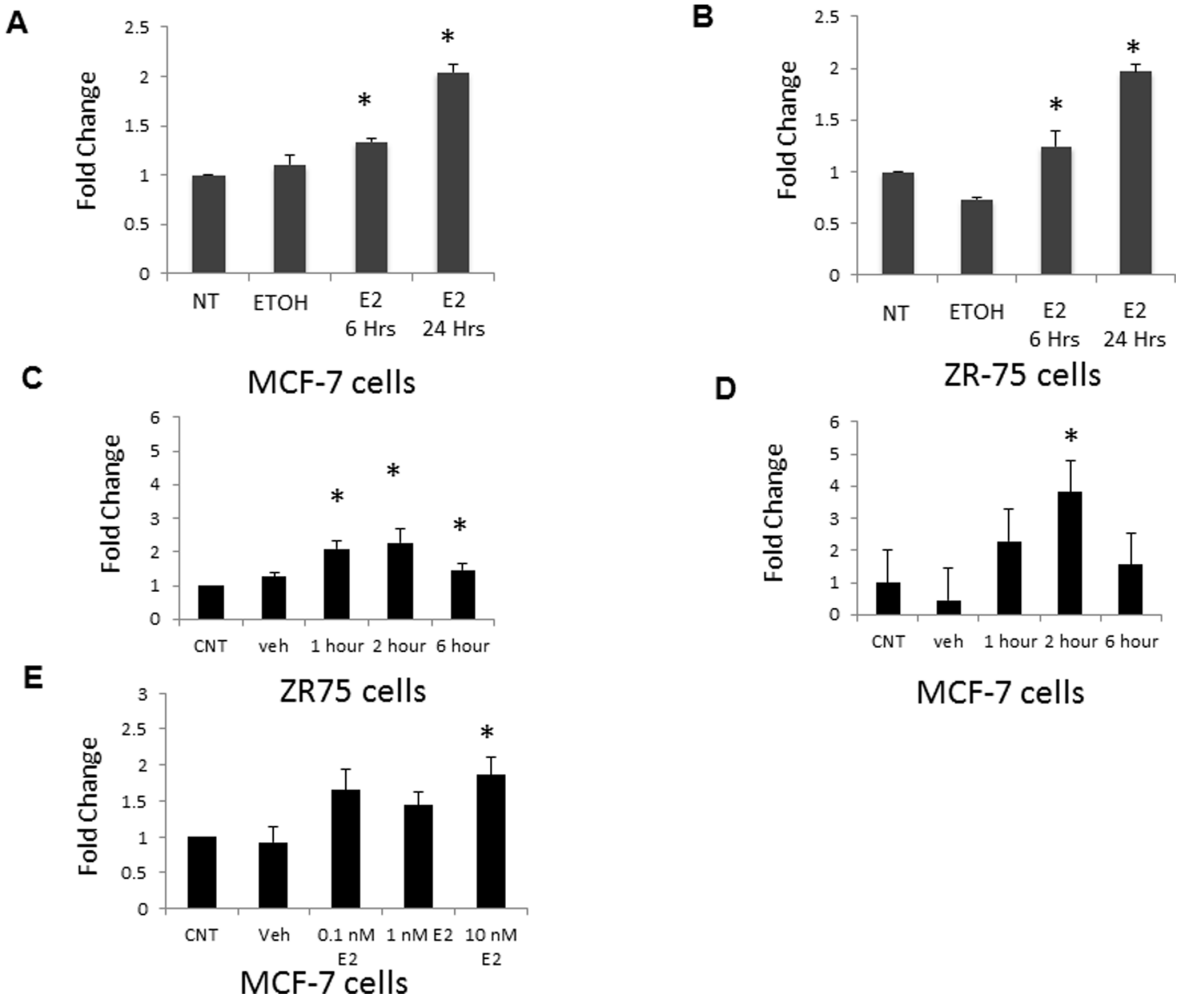
Estrogen increases Mcl-1 mRNA expression. Real-time PCR analysis of Mcl-1 transcript levels in (A) MCF-7 and (B) ZR-75 was performed following 24-hour stimulation with estrogen (10 nM). In addition, Real-time PCR analysis of Mcl-1 transcript levels in (C) MCF-7 and (D) ZR-75 was performed following a short 6-hour time course with estrogen (10 nM). E) Finally, MCF-7 cells were treated with a range of estrogen concentrations (0.1 nM–10 nM) and Mcl-1 mRNA levels analyzed after 6 hours. In all experiments, 100 ng template RNA was amplified using primers specific to Mcl-1. qPCR results were standardized using primers for housekeeping gene cyclophilin or TATA box binding protein (TBP). Fold change represents the results relative to changes in basal levels observed in untreated sample. Data represents the mean of three independent experiments ± standard error. (* indicates p≤0.0002 compared to untreated control cells).

We evaluated whether estrogen is involved in regulating Mcl-1 transcription. We used two ERα expressing breast cancer cell lines, MCF-7 and ZR-75 (Figure S1 in File S1), as models of ERα+ breast cancer and treated with estrogen (10 nM). We isolated total RNA at both 6 hours and 24 hours post estrogen treatment. We detected Mcl-1 mRNA levels by quantitative real-time PCR. We found that mRNA expression increased approximately 1.5-fold in both MCF-7 and ZR-75 cells after 6 hours of estrogen treatment, (Figure 1 A,B) and increased 2-fold in both MCF-7 and ZR-75 cells after 24-hours after estrogen treatment (Figure 1 A, B). This increase in Mcl-1 mRNA was evident as early as 1 hour following estrogen treatment in both MCF-7 and ZR-75 cells (Figure 1 C, D). In addition, we treated MCF-7 cells with increasing doses of estrogen (0.1 nM to 10 nM). We found that mRNA levels increased to 2 fold at 10 nM estrogen treatment after six hours. Control cells (untreated and EtOH (vehicle control)) failed to show a similar increase (Figure 1). In ERα negative breast cancer cell lines MDA MB 231 and SKBr3 (Figure S1 in File S1), the amount of Mcl-1 mRNA levels failed to increase (Figure S2 in file S1). This suggests that in ERα+ breast cancer cells, estrogen signaling is involved in up-regulating Mcl-1 transcription.

**Figure 2:**
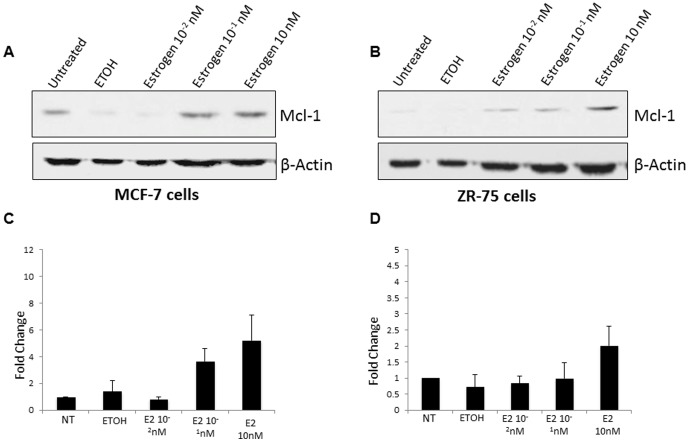
Estrogen increases Mcl-1 protein expression in ERα+ breast cancer cell lines. (A) Western blot analysis of MCF-7 was performed following 24-hour stimulation with increasing concentrations of estrogen (10^−2^ nM–10 nM). (B) Western blot analysis of ZR-75 was performed following 24-hour stimulation with increasing concentrations of estrogen (10^−2^ nM–10 nM). In both experiments, cells were serum-starved for 5 days prior to treatment with estrogen. Blots were reprobed with anti-β-actin as a loading control. (C) Relative accumulation of Mcl-1 protein expression in MCF-7 cells was confirmed by densitometry. Data represents mean of three independent experiments ± standard error. (D) Relative accumulation of Mcl-1 protein expression in ZR-75 cells was confirmed by densitometry. Data represents mean of three independent experiments ± standard error.

To determine the role of estrogen in regulating Mcl-1 protein expression, we treated MCF-7 and ZR-75 cells with increasing concentrations of estrogen and evaluated total protein levels. We treated the cell lines with a range of estrogen concentrations (10^−2^ nM–10 nM) for 24 hours. In both MCF-7 and ZR-75 cells, we found the highest increase in Mcl-1 protein expression at 10 nM estrogen treatment (Figure 2A–B). We normalized these values using densitometry and found at 10 nM of estrogen, there was a 5-fold and 2.5-fold increase in MCF-7 and ZR-75 cells, respectively (Figure 2C–D). Control cells (untreated and vehicle control) failed to show a similar increase (Figure 2C–D). As a positive control for estrogen treatment, progesterone receptor expression levels were shown to increase after estrogen treatment in MCF-7 cells (Figure S1 in File S1) This data suggests that estrogen signaling is involved in up-regulating Mcl-1 protein expression in ERα+ breast cancer cell lines.

### Estrogen treatment does not increase Mcl-1 protein expression in ERα- cell lines

**Figure 3:**
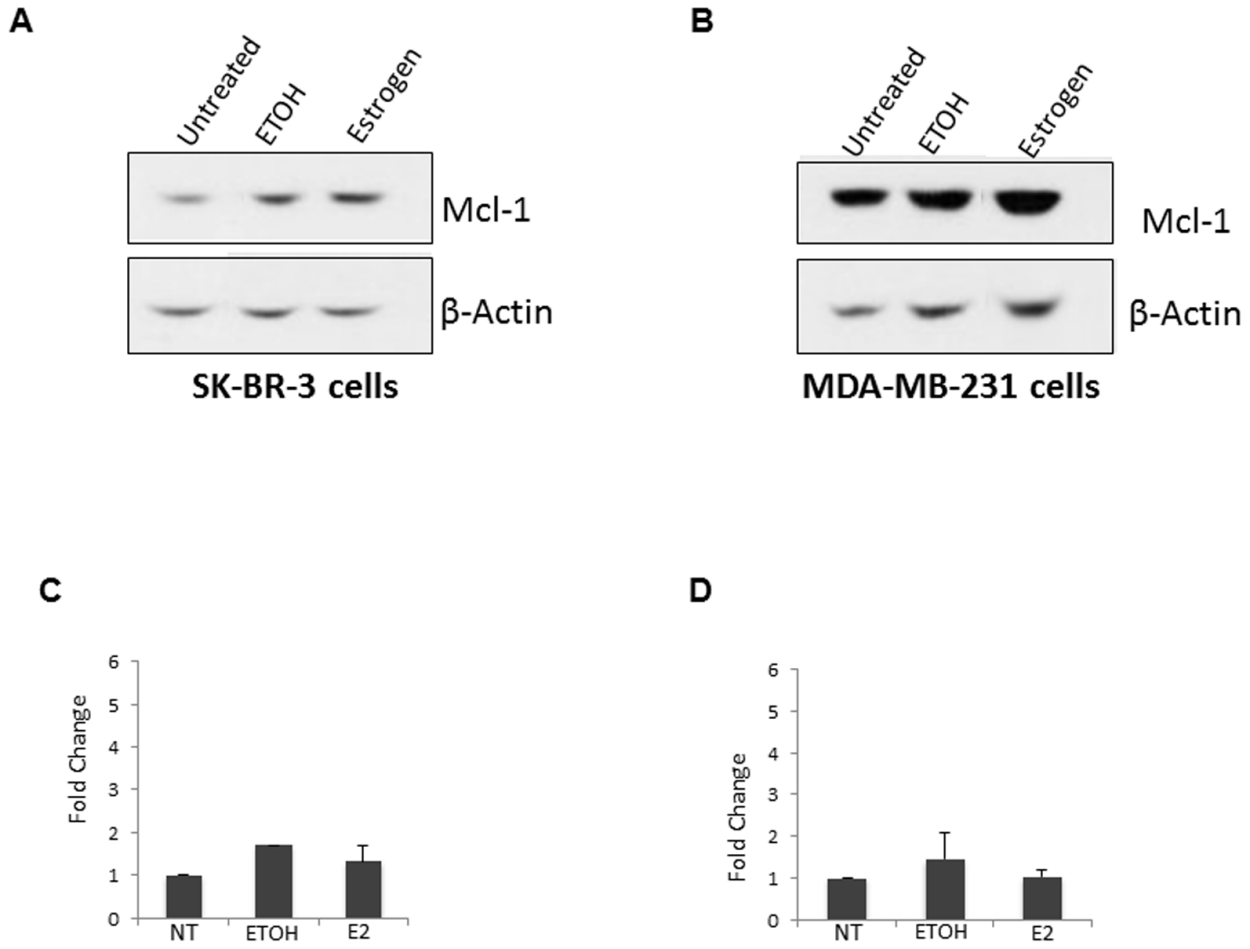
Estrogen fails to increase Mcl-1 protein expression in ERα- breast cancer cell lines. (A) Western blot analysis of SK-BR-3 cells was preformed following 24-hour stimulation with estrogen (10 nM). (B) Western blot analysis of MDA-MB-231 cells was preformed following 24-hour stimulation with estrogen (10 nM). In both experiments, cells were serum-starved for 5 days prior to treatment with estrogen. Blots were reprobed with anti-β-actin as a loading control. (C) Relative accumulation of Mcl-1 protein expression in SK-BR-3 cells, confirmed by densitometry. Data represents mean of two independent experiments ± standard error. (D) Relative accumulation of Mcl-1 protein expression in MDA-MB-231 cells, confirmed by densitometry. Data represents mean of two independent experiments ± standard error.

Estrogen seems to regulate Mcl-1 expression at both the protein and mRNA level in ERα+ breast cancer cell lines, suggesting that ER mediates this expression. To determine the role of ER, we evaluated the effect of estrogen on Mcl-1 expression in two ERα- breast cancer cell lines, SK-BR-3 and MDA-MB-231 cells. SK-BR-3 do not express ERα, however, they do express ERβ alone. MDA-MB-231cells does not express ERα or ERβ [16], [17]. We treated these cell lines with estrogen (10 nM). After 24 hours, we failed to detect an increase in Mcl-1 protein expression in either SK-BR-3 or MDA-MB-231cells (Figure 3 A, B). Using densitometry, we found a negligible fold-change in Mcl-1 expression after estrogen treatment when compared to both an untreated and vehicle control (Figure 3 C, D). Overall, this data suggests that estrogen signaling requires the presence of ERα to increase Mcl-1 expression.

### Estrogen antagonists Tamoxifen and Fulvestrant decrease Mcl-1 expression at both the protein and mRNA level

**Figure 4:**
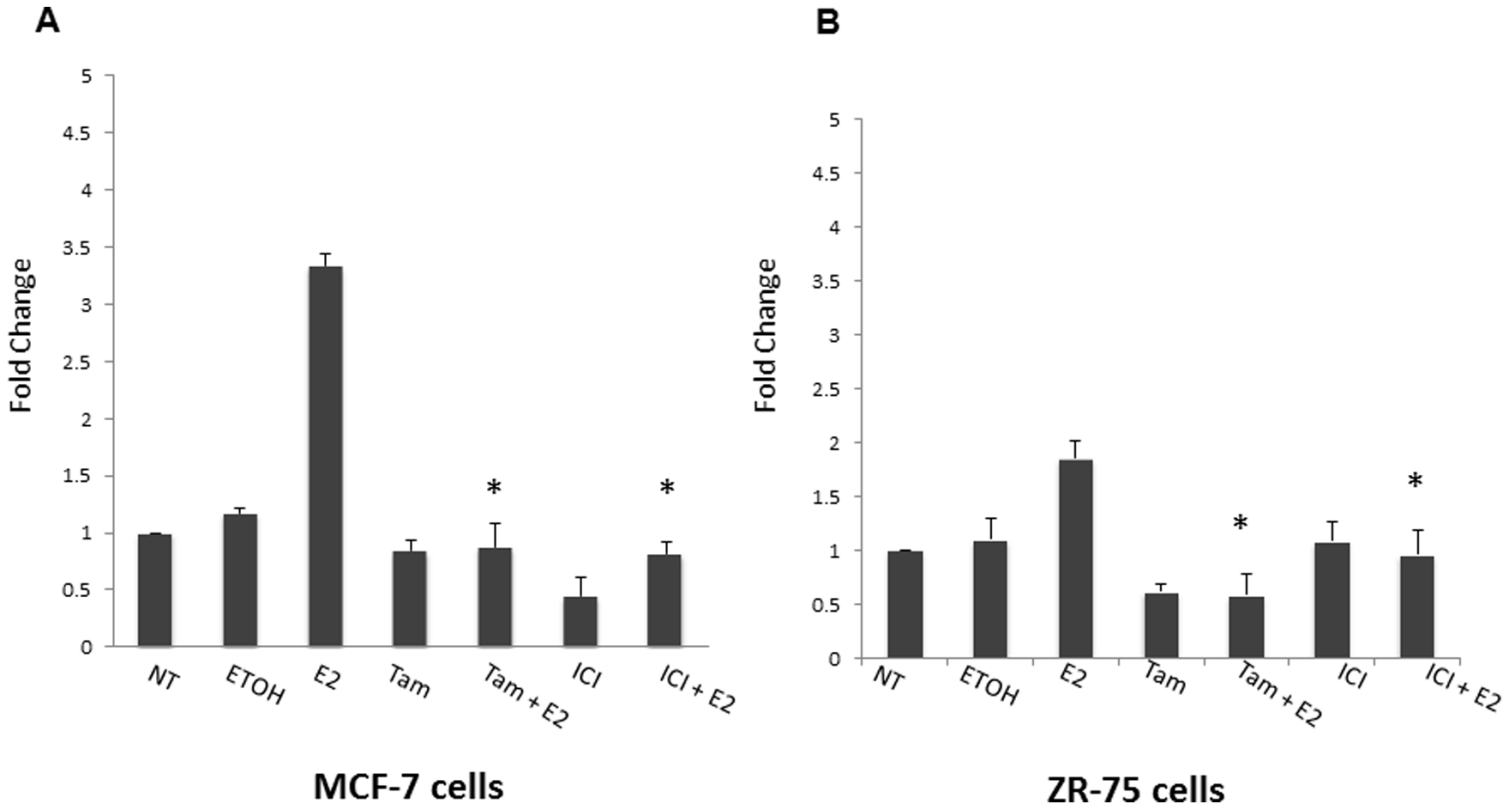
Anti-estrogens Tamoxifen and Fulvestrant decrease Mcl-1 mRNA expression. (A) Real-time PCR analysis of Mcl-1 transcript levels in MCF-7 was determined following 24-hour treatment with Tamoxifen (Tam, 200 nM) or Fulvestrant (ICI, 500 nM) in combination with estrogen (E2, 10 nM). (B) Real-time PCR analysis of Mcl-1 transcript levels in ZR-75 was determined following 24-hour treatment with Tamoxifen (Tam, 200 nM) or Fulvestrant (ICI, 500 nM) in combination with estrogen (E2, 10 nM). In both experiments 100 ng template RNA was amplified using primers specific to Mcl-1. qPCR results were standardized using primers for housekeeping gene cyclophilin. Results are expressed as fold change relative to changes in basal levels observed in untreated sample. Data represents the mean of three independent experiments ± standard error. (* indicates p≤0.0001 compared to untreated control cells; # indicates p≤0.002 compared to untreated control cells).

**Figure 5:**
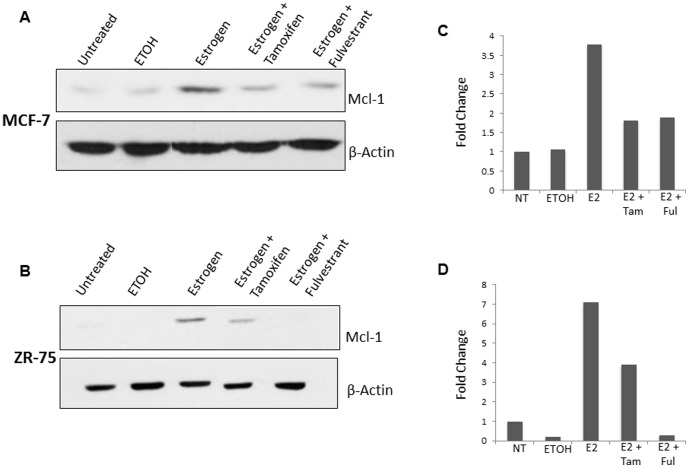
Treatment with anti-estrogen Tamoxifen and Fulvestrant decrease Mcl-1 protein expression. (A) Western blot analysis of MCF-7 cells was performed following 24-hour treatment with either Tamoxifen (200 nM) or Fulvestrant (500 nM) in combination with estrogen (10 nM). (B) Western blot analysis of ZR-75 cells was performed following 24-hour hour treatment with either Tamoxifen (200 nM) or Fulvestrant (500 nM) in combination with estrogen (10 nM). Blots were reprobed with anti-β-actin as a loading control. (C) Relative accumulation of Mcl-1 protein expression in (C) MCF-7 cells, and (D) ZR-75 cells confirmed by densitometry. This represents the trend in three independent experiments.

Estrogen antagonists are used in breast cancer therapy to block estrogen receptor activation. We determined whether estrogen regulates Mcl-1 expression through a ligand-dependent mechanism involving ERα activation. We used two anti-estrogens, Tamoxifen and Fulvestrant (ICI), which antagonize the ER by inhibiting estrogen binding to ERα [18], [19]. We treated MCF-7 and ZR-75 cells with either Tamoxifen (200 nM) or Fulvestrant (500 nM) in combination with estrogen (10 nM) for 24 hours and compared these findings to cells treated with estrogen, Tamoxifen or Fulvestrant alone. In MCF-7 cells, we found that estrogen treatment resulted in a 3.5-fold increase in RNA expression, whereas both Tamoxifen and Fulvestrant treatment in combination with estrogen failed to increase mRNA levels (Figure 4A). In ZR-75 cells, we found that estrogen treatment resulted in a 2-fold increase in Mcl-1 mRNA expression whereas both Tamoxifen and Fulvestrant treatment in combination with estrogen failed to increase Mcl-1 expression (Figure 4B). These results were compared to both an untreated and vehicle control, which showed a no significant increase (Figure 5). In addition, we studied the effects of Tamoxifen and Fulvestrant on Mcl-1 expression at the protein level. In both MCF-7 and ZR-75 cell lines, Tamoxifen and Fulvestrant treatment in combination with estrogen resulted in a decrease in Mcl-1 protein expression (Figure 5 A, B). This data was normalized using densitometry, which showed a reduction of approximately 1.8-fold in MCF-7 cells after both Tamoxifen and Fulvestrant treatment (Figure 5C). In ZR-75 cells, a reduction of 3-fold and 6.5-fold was seen after Tamoxifen and Fulvestrant treatment, respectively (Figure 5D). Taken together, Tamoxifen and Fulvestrant treatment results in a decrease in Mcl-1 expression at both the protein and mRNA level.

### Knockdown of ERα decreases Mcl-1 mRNA expression

**Figure 6:**
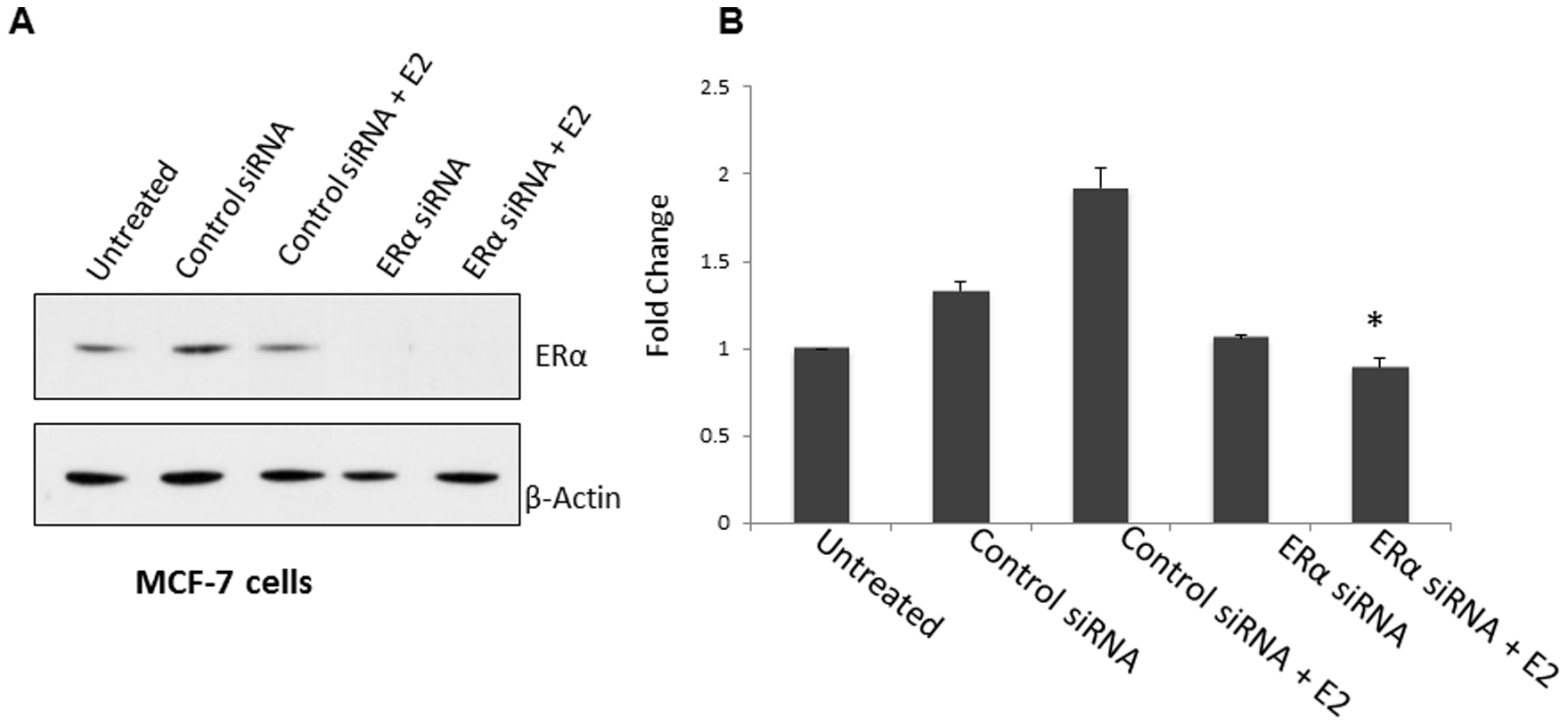
Knockdown of ERα results in decrease in Mcl-1 mRNA expression. (A) Western blot analysis confirms ERα silencing in MCF-7 cells after siRNA transfection. Blot was reprobed with anti-β-actin as a loading control. (B) Real-time PCR analysis of Mcl-1 transcript levels in MCF-7 cells was performed. Cells were serum starved for 5 days prior to transfection. After transfection, cells were stimulated with estrogen (E2, 10 nM) for 24-hours. For qPCR, 100 ng template RNA was amplified using primers specific to Mcl-1. qPCR results were standardized using primers for housekeeping gene cyclophilin. Results are expressed as fold change relative to changes in basal levels observed in untreated sample. Data represents the mean of three independent experiments ± standard error. (* indicates p≤0.001 compared to untreated control cells).

To investigate the role of ERα in regulating Mcl-1 expression, we performed a knockdown of ERα in MCF-7 cells using siRNA. Serum-starved MCF-7 cells were transfected with either a pool of 3 small interfering (si)RNAs against ERα or control siRNA. Silencing of ERα was confirmed by western blot analysis (Figure 6A). After transfection, cells were treated with estrogen (10 nM) over a 24-hour period. Knockdown of ERα blocked estrogen induced Mcl-1 mRNA expression (Figure 6B). This was compared to control siRNA, which resulted in a 2-fold increase in Mcl-1 mRNA expression when treated with estrogen (Figure 6B). Overall, this suggests that ERα plays an important role in regulating Mcl-1 mRNA expression.

### Sequence analysis of Mcl-1 promoter reveals presence of 5 ERE half-sites

**Figure 7:**
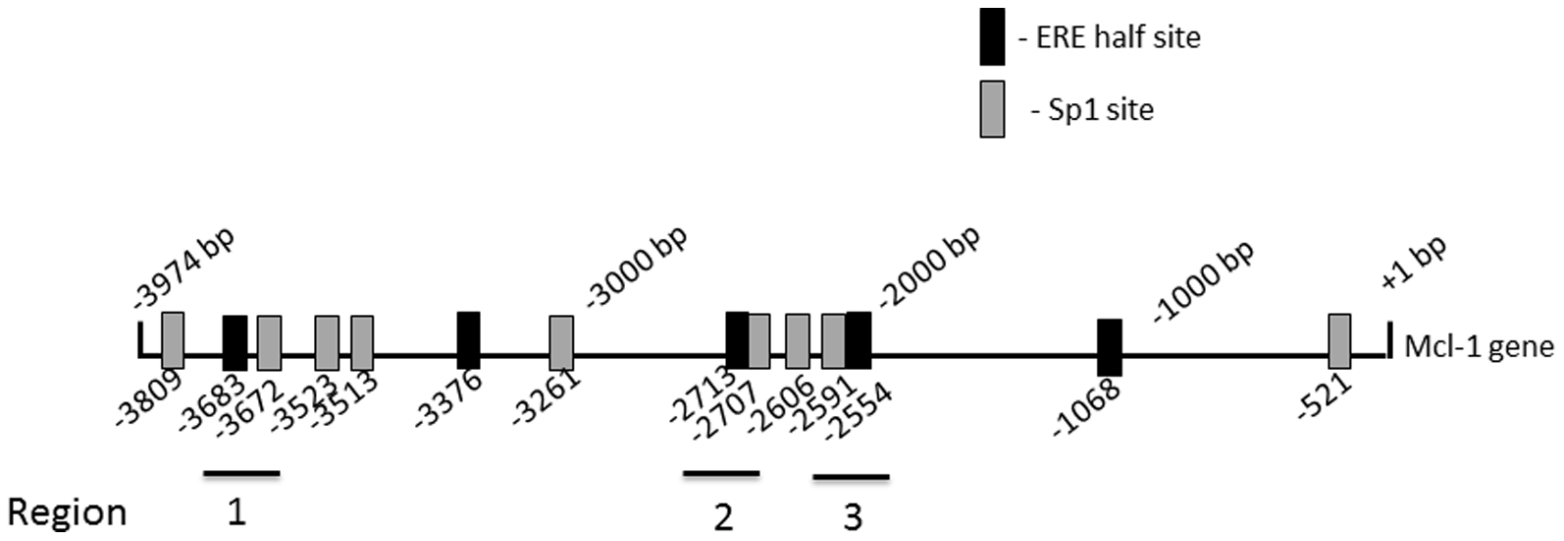
Schematic representation of *Mcl-1* gene showing approximate locations of ERE half-sites and Sp1 binding sites. A sequence analysis of the Mcl-1 promoter region showed that the Mcl-1 promoter region includes 5 ERE half-sites (black boxes). These half-sites are located at 3683 bp, 3376 bp, 2713 bp, 2554 bp and 1068 bp upstream of the translation start site. In addition, there are multiple Sp-1 transcription factor sites within the promoter (gray boxes). Three of the ERE half-sites, located at 3683 bp, 2713 bp and 2554 bp upstream of the translation start site, are in close proximity to Sp1 binding sites. These regions (regions 1–3) are denoted by black lines.

To evaluate whether Mcl-1 expression is regulated by ER binding to the Mcl-1 promoter, we performed a sequence analysis of the Mcl-1 promoter region. As shown in Figure 7, the Mcl-1 promoter region includes 5 ERE half-sites. These half-sites are located at 3683 base pairs (bp), 3376 bp, 2713 bp, 2554 bp and 1068 bp upstream of the translation start site (Figure 7). In addition, we identified multiple Sp-1 transcription factor sites within the promoter (Figure 7). Three of the ERE half-sites, located at 3683 bp, 2713 bp and 2554 bp upstream of the translation start site, are in close proximity to Sp1 binding sites denoted as regions 1–3 (Figure 7). Half ERE sites located beside a Sp1 site are considered to be potential ER binding sites in complex with Sp1, leading to gene expression. This sequence analysis suggests that estrogen may regulate Mcl-1 expression through a mechanism involving ER and Sp1 binding within the Mcl-1 promoter.

### ERα binds to the Mcl-1 promoter

**Figure 8:**
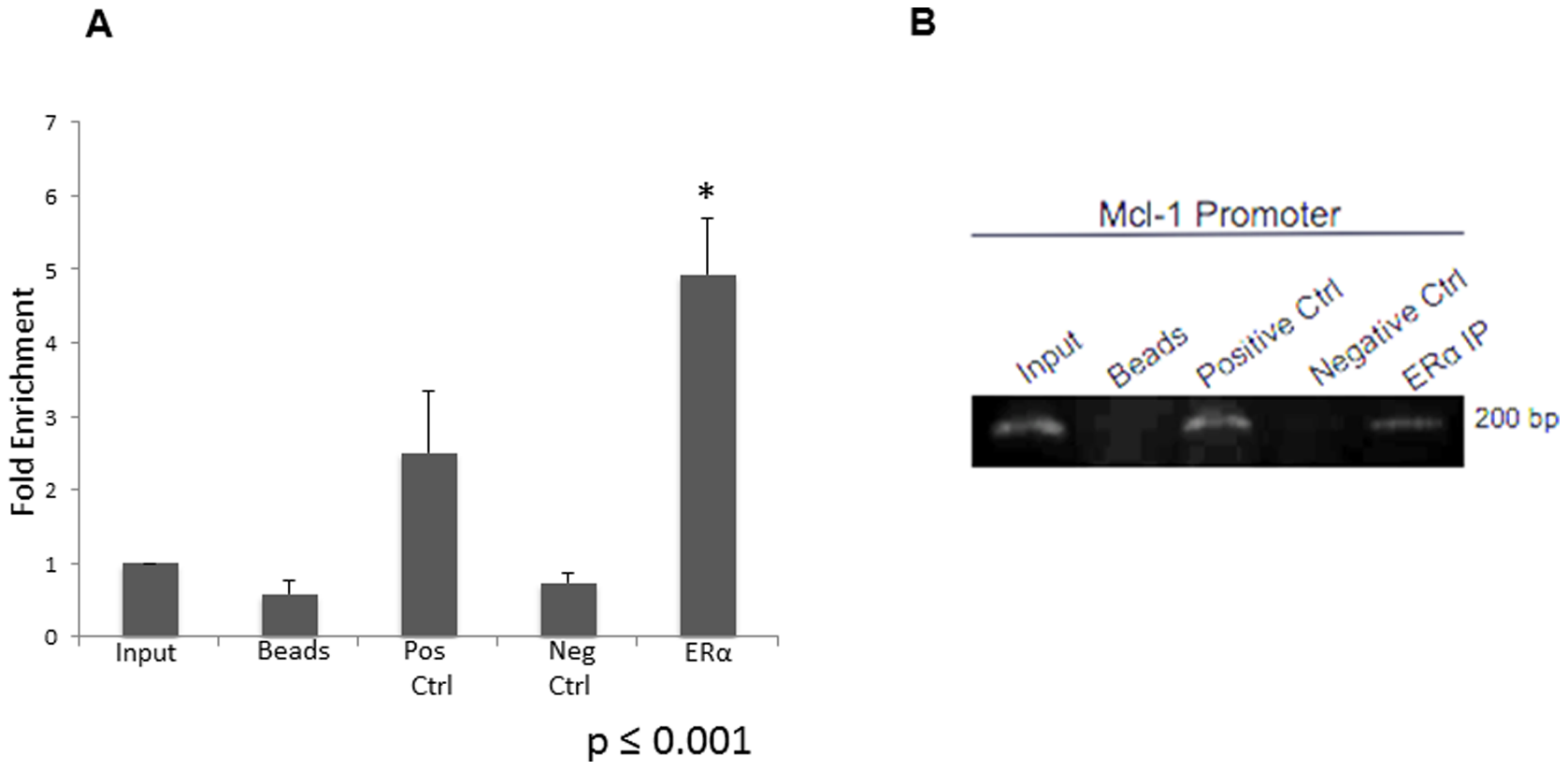
ERα binds to specific region within Mcl-1 promoter in MCF-7 cells. (A) Chromatin immunoprecipitation (ChIP) was performed using antibody specific to ERα. ChIP was performed 6 hours after estrogen (10 nM) treatment. Positive control (immunoprecipitation of RNA Polymerase II), negative control (immunoprecipitation of Normal Mouse IgG) and no antibody control (beads alone) are shown. Results represent fold enrichment values obtained by comparing cT values of ChIP samples to cT values of input. Data represents the mean of 3 independent experiments ± standard error. * indicates p≤0.001 compared to input; # indicates p≤0.2 compared to input. (B) PCR products were run on agarose gel to evaluate ChIP specificity. Primers specific to ERE half-site located 3683 bp upstream of translation start site of Mcl-1 were used.

To evaluate whether ERα binds to the Mcl-1 promoter, a chromatin immunoprecipitation (ChIP) experiment was conducted with ERα+ MCF-7 cells. To assess ERα binding, primers directed to region 1 containing an ERE half site and Sp1 transcription factor site were designed (Figure 7). MCF-7 cells were treated with estrogen (10 nM) and ChIP was performed 6 hours post-treatment. ChIP was performed using antibodies directed against ERα. After stimulation with estrogen, ERα was detected on the Mcl-1 promoter at both 6 hours (Figure 8) and 24 hours (data not shown) after estrogen treatment. After 6 hours, estrogen treatment resulted in a 5-fold enrichment of ERα at the Mcl-1 promoter (Figure 8A). Both the negative control (Normal Mouse IgG) and no antibody (beads alone) control had negligible fold-enrichment values, suggesting that they failed to immunoprecipitate Mcl-1 promoter fragments (Figure 8). We also found that the Mcl-1 promoter containing the ERE half sites in a luciferase reporter gene results in increased luciferase activity following estrogen treatment (Figure S3 in file S1). In a control experiment, a ChIP was performed following immunoprecipitation of ER α using primers for the non-estrogen responsive gene cyclophilin. There was no binding detected (Figure S4 in file S1). Overall, this suggests that ERα is involved in regulating Mcl-1 expression by binding to an ERE site(s) within the Mcl-1 promoter.

### Estrogen increases ERα and Sp1 binding to the Mcl-1 promoter

**Figure 9:**
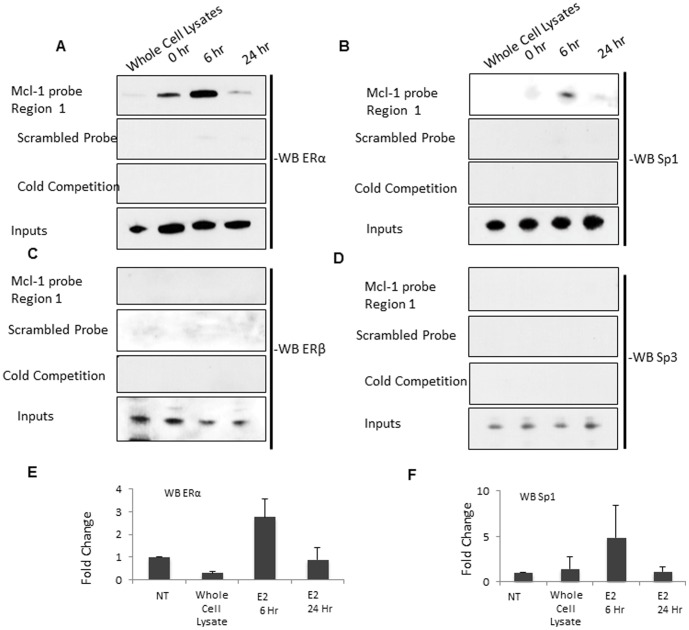
Estrogen increases ERα binding to specific region on Mcl-1 promoter. (A) Streptavidin pull-down assay to detect ER and transcription factor binding to a 50 bp double-stranded biotin labeled probe specific to Mcl-1 promoter region of interest (region 1). Cells were stimulated with estrogen (10 nM) and nuclear extracts were taken 6 and 24-hours post-estrogen treatment. Pull-down products were analyzed using SDS/polyacrylamide gel electrophoresis and western blotting. Both a scrambled probe and an excess of unlabeled probe were used as a control. Blot was probed with antibody specific for ERα. (B) Blot was probed with antibody specific for ERβ. (C) Blot was probed with antibody specific for Sp1. (D) Blot was probed with antibody specific for Sp3. (E) Relative accumulation of ERα protein expression, confirmed by densitometry. (F) Relative accumulation of Sp1 protein expression, confirmed by densitometry. (G) Schematic representation of *Mcl-1* gene showing approximate locations of biotin labeled probes used for Streptavidin pull-down.

**Figure 10:**
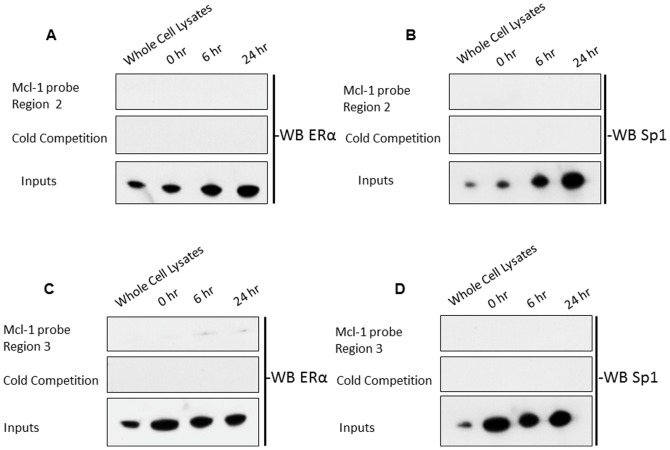
Estrogen receptor fail to bind to other ERE half sites in the Mcl-1 promoter. Streptavidin pull-down assay to detect ER and transcription factor binding to a 50 bp double-stranded biotin labeled probe specific to Mcl-1 promoter region of interest (region 2). Cells were stimulated with estrogen (10 nM) and nuclear extracts were taken 6 and 24-hours post-estrogen treatment. Pull-down products were analyzed using SDS/polyacrylamide gel electrophoresis and western blotting. Both a scrambled probe and an excess of unlabeled probe were used as a control. Blot was probed with antibody specific for ERα. (B) Blot was probed with antibody specific for Sp1. (C) Streptavidin pull-down assay to detect ER and transcription factor binding to a 50 bp double-stranded biotin labeled probe specific to Mcl-1 promoter region of interest (region 3). Cells were stimulated with estrogen (10 nM) and nuclear extracts were taken 6 and 24-hours post-estrogen treatment. Both a scrambled probe and an excess of unlabeled probe were used as a control. Blot was probed with antibody specific for ERα. (D) Blot was probed with antibody specific for Sp1.

After demonstrating that ERα binds to the Mcl-1 promoter using ChIP, we further validated this by performing a streptavidin pull-down assay. We designed a 50 bp biotin-labeled probe that was complementary to region 1, a half ERE site that is 3683 bp (region 1) upstream of the translation start site (Figure 7). We treated ERα+ MCF-7 cells with estrogen and performed nuclear extraction at both 6 hours and 24 hours post-treatment. The Mcl-1 promoter specific probe was able to pull down ERα at 6 hours post-estrogen treatment (Figure 9A). We quantified this data using densitometry and showed that there is a 2.5-fold increase in ERα expression at 6-hours post-estrogen treatment (Figure 9E). We further validated this data using a scrambled probe, which was unable to pull down ERα (Figure 9A). In addition, cold competition was performed using an excess of unlabeled probe. With cold competition, we were able to successfully compete away ERα (Figure 9A). We also evaluated ERβ binding, however, the Mcl-1 promoter specific probe was unable to pull down ERβ after both 6 hours and 24 hours of estrogen treatment (Figure 9B). Since the ERE site under study is a half-site, we were interested in determining whether transcription factors Sp1 or Sp3 are also involved in regulating Mcl-1 expression. Upon evaluation with antibodies against Sp1 and Sp3, the Mcl-1 promoter specific probe was able to pull down Sp1 but not Sp3 at 6-hours post-estrogen treatment (Figures 9C and 9D). Densitometry showed a 5-fold increase in Sp1 expression at 6 hours post-estrogen treatment (Figure 9F). We also studied two other regions within the Mcl-1 promoter that contained ERE half sites. These regions are located 2713 bp (region 2) and 2554 bp (region 3) upstream of the translation start site (Figure 7). Using 50 bp biotin-labeled probes directed to these sites, we were unable to pulldown ERα, or Sp1 to these regions (Figure 10). Overall, these results suggest that estrogen is involved in regulating Mcl-1 expression through a mechanism involving ERα and Sp1 binding to a specific ERE half-site and Sp1 site that is approximately 3683 bp upstream of the translation start site within the Mcl-1 promoter.

## Discussion

Our results indicate that estrogen is involved in up-regulating Mcl-1 expression specifically through ERα. We propose that ERα binds to a specific ERE half-site at 3683 bp upstream of the *Mcl-1* gene translation start site in complex with a Sp1. Once bound to the Mcl-1 promoter, this ERα-Sp1 complex is capable of mediating gene transcription and up-regulates Mcl-1 expression in the presence of estrogen. This suggests that ERα in complex with Sp1 binds to the Mcl-1 promoter in a ligand-dependent mechanism involving estrogen (Figure S5 in file S1).

Our findings suggest that ERα is involved in up-regulating Mcl-1 expression by binding to a specific half ERE site in complex with Sp1 sites within the Mcl-1 promoter. This is in agreement with previous literature, as estrogen has been shown to mediate gene transcription through a complex involving ERα and Sp1 [20]. For example, Wu-Peng et al. (1992) found that in order for estrogen to activate creatine kinase B, a complex between Sp1 and an ER DNA-binding domain was required [21]. Additionally, Dubik and Shiu (1992) identified an interaction between Sp1 and an ERE half-site required for the activation of c-myc [22]. Subsequently, many studies have found numerous interactions between GC-rich Sp1 binding domains and ERE half-sites required for estrogen-mediated activity [20], [23].

Mcl-1 is an anti-apoptotic member of the Bcl-2 protein family, which are involved in the mitochondrial-mediated intrinsic pathway of apoptosis [3]. Mcl-1 has a very short half-life, indicating that tight regulation at both the transcriptional and translational level is required [3]. At the transcriptional level, Mcl-1 is regulated by several transcription factors, such as members of the STAT family and Elk-1 [3], [6]. Additionally, Mcl-1 is modified post-transcriptionally by alternative splicing that results in two Mcl-1 isoforms, Mcl-1_L_ and Mcl-1_s_, which have opposing roles in regulating cell death [24]. Mcl-1 is also regulated at the translational level by several mechanisms controlling both caspase and proteasomal degradation [3]. Post-translational modifications, such as phosphorylation and ubiquitination, also allow for tight regulation of Mcl-1 stability and degradation [3]. In breast cancer, the *Mcl-1* gene is frequently amplified, allowing for its overexpression despite its short half-life [5]. However, Mcl-1 expression is tightly controlled by both proteasomal and caspase-dependent degradation pathways [25]. Therefore, malignant cells must develop alternative mechanisms to overcome Mcl-1's rapid degradation. Our results indicate that ERα is an important regulator in maintaining Mcl-1 expression and may counteract post-translational Mcl-1 degradation, allowing for evasion of apoptosis in ERα positive breast cancer.

Many studies have indicated that crosstalk between estrogen and growth factor signaling pathways may promote drug resistance to hormonal therapy [2]. Previous literature has demonstrated that Mcl-1 is a downstream target of EGF in many different types of cancer, including breast cancer [6], [7]. Further studies have shown that estrogen may be involved in up-regulating signaling pathways that are associated with EGF, such as the MAPK or PI3K/AKT pathways [26]. EGF may initiate signaling cascades that phosphorylate and activate AF1 sites within ERα, contributing to Tamoxifen resistance [27], [28]. Estrogen may up-regulate important components of EGF-mediated signaling cascades, such as activating MAPK protein Erk in a mechanism involving ERα in MCF-7 cells [2]. The cross-talk between EGF and estrogen on Mcl-1 expression will be the focus of future investigation.

Estrogen has been implicated in regulating several other members of the Bcl-2 family of proteins. Bcl-2 is an anti-apoptotic protein that is frequently overexpressed in cancer [5]. Several studies have indicated that estrogen may up-regulate Bcl-2, allowing for evasion of apoptosis [29], [30]. Estrogen-mediated overexpression of Bcl-2 is regulated by ERα, as both Tamoxifen and Fulvestrant can decrease Bcl-2 expression [31]. In addition, estrogen may be involved in regulating pro-apoptotic BH3-only protein Noxa, which is associated with regulating Mcl-1 expression [32]. While the role of estrogen in regulating Bcl-x_L_ expression in breast cancer remains unknown, a study with cultured hippocampal neurons demonstrated that estrogen increases Bcl-x_L_ expression through a mechanism involving an ERE site within the *Bcl-x_L_* gene [33].

ERα-mediated overexpression of Mcl-1 may contribute to drug resistance by providing a mechanism by which breast cancer cells can evade apoptosis. Under survival conditions, Mcl-1 sequesters BH3-only protein Noxa, preventing it from binding to pro-apoptotic proteins Bax and Bak [3]. This action prevents the loss of membrane potential, production of reactive oxygen species, and release of mitochondrial protein cytochrome c, which are required for initiation of apoptosis [3]. Our results suggest that estrogen up-regulates Mcl-1 expression, allowing for cell survival. Recently, small molecular inhibitors against Bcl-2 family members, such as the drugs ABT-737 and obatoclax, have shown promise in combatting drug resistance in breast cancer [34]–[36]. Furthermore, a small molecular inhibitor specifically targeting Mcl-1, called maritoclax, has been developed and has shown effectiveness in combating resistance to ABT-737 treatment [37]. Therefore, a better understanding of estrogen-mediated Mcl-1 up-regulation may allow for better targeted therapies for breast cancer patients.

## Conclusions

Overall, Mcl-1 expression appears to be regulated through a mechanism involving ERα, possibly through a complex with Sp1. Estrogen treatment up-regulates Mcl-1 expression, conceivably providing a mechanism of drug resistance in hormonal therapy. Future studies regarding the role of estrogen in mediating apoptosis will help determine whether Mcl-1 is a valid molecular target for breast cancer therapy.

## Supporting Information

File S1 **Figure S1 Estrogen receptor, and Progestrone receptor expression in breast cancer cell lines.** A) MCF-7, ZR75, SkBr3 and MDA MB 231 cells were lysed and western blotted for estrogen receptor, progesterone receptor and Mcl-1. Loading control was actin. B) MCF-7 cells were treated with estrogen (10 nM) for 24 hours and western blotted for progesterone receptor (isoforms A and B), Mcl-1 was also western blotted and actin as a loading control. Ethanol (ETOH) was used as a vehicle control and untreated cells as a negative control. **Figure S2 Mcl-1 mRNA levels after estrogen treatment in MDA MB 231 and SKBr3 cells.** Real-time PCR analysis of Mcl-1 transcript levels in (A) MDA MB 231 and (B) SKBr3 cells was performed following 24-hour stimulation with estrogen (10 nM). In all experiments, 100 ng template RNA was amplified using primers specific to Mcl-1. qPCR results were standardized using primers for housekeeping gene TATA box binding protein (TBP). Fold change represents the results relative to changes in basal levels observed in untreated sample. Data represents the mean of three independent experiments ± standard error. (* indicates p≤0.0002 compared to untreated control cells). **Figure S3 Estrogen activates the Mcl-1 promoter.** The 4 kb Mcl-1 promoter containing ERE half sites was cloned in a luciferase reporter gene construct. The construct was transfected into MCF-7 cells and the cells were either treated with ETOH (vehicle control) or estrogen for 6 hours. The luciferase activity was detected using previously published protocols. **Figure S4. ChIP analysis using off-target cyclophillin gene.** Chromatin immunoprecipitation (ChIP) was performed using antibody specific to ERα. ChIP was performed 6 hours after estrogen (10 nM) treatment. Positive control (immunoprecipitation of RNA Polymerase II), negative control (immunoprecipitation of Normal Mouse IgG) and no template control are shown. The PCR products were run on agarose gel to evaluate ChIP specificity. Primers specific to cyclophilin promoter were used as an off-target control. **Figure S5 Proposed mechanism of ERα-mediated Mcl-1 regulation.** We propose that ERα binds to a specific ERE half-site at 3683 bp upstream of the translation start site in complex with a GC-rich Sp1 binding domain within the promoter of Mcl-1. Once bound to the promoter, this ERα-Sp1 complex is capable of mediating gene transcription and up-regulates Mcl-1 expression in the presence of estrogen.
(PPTX)
