# Supplementary material for: Estrogen Regulation of Anti-Apoptotic Bcl-2 Family Member Mcl-1 Expression in Breast Cancer Cells
Source: PLoS One. 2014 Jun 27;9(6):e100364. doi: 10.1371/journal.pone.0100364 (PMC4074091; doi:10.1371/journal.pone.0100364)
Supplement: File S1 — Figure S1 Estrogen receptor, and Progestrone receptor expression in breast cancer cell lines. A) MCF-7, ZR75, SkBr3 and MDA MB 231 cells were lysed and western blotted for estrogen receptor, progesterone receptor and Mcl-1. Loading control was actin. B) MCF-7 cells were treated with estrogen (10 nM) for 24 hours and western blotted for progesterone receptor (isoforms A and B), Mcl-1 was also western blotted and actin as a loading control. Ethanol (ETOH) was used as a vehicle control and untreated cells as a negative control. Figure S2 Mcl-1 mRNA levels after estrogen treatment in MDA MB 231 and SKBr3 cells. Real-time PCR analysis of Mcl-1 transcript levels in (A) MDA MB 231 and (B) SKBr3 cells was performed following 24-hour stimulation with estrogen (10 nM). In all experiments, 100 ng template RNA was amplified using primers specific to Mcl-1. qPCR results were standardized using primers for housekeeping gene TATA box binding protein (TBP). Fold change represents the results relative to changes in basal levels observed in untreated sample. Data represents the mean of three independent experiments ± standard error. (* indicates p≤0.0002 compared to untreated control cells). Figure S3 Estrogen activates the Mcl-1 promoter. The 4 kb Mcl-1 promoter containing ERE half sites was cloned in a luciferase reporter gene construct. The construct was transfected into MCF-7 cells and the cells were either treated with ETOH (vehicle control) or estrogen for 6 hours. The luciferase activity was detected using previously published protocols. Figure S4. ChIP analysis using off-target cyclophillin gene. Chromatin immunoprecipitation (ChIP) was performed using antibody specific to ERα. ChIP was performed 6 hours after estrogen (10 nM) treatment. Positive control (immunoprecipitation of RNA Polymerase II), negative control (immunoprecipitation of Normal Mouse IgG) and no template control are shown. The PCR products were run on agarose gel to evaluate ChIP specificity. Primers specific [file pone.0100364.s001.pptx]

## Slide 1
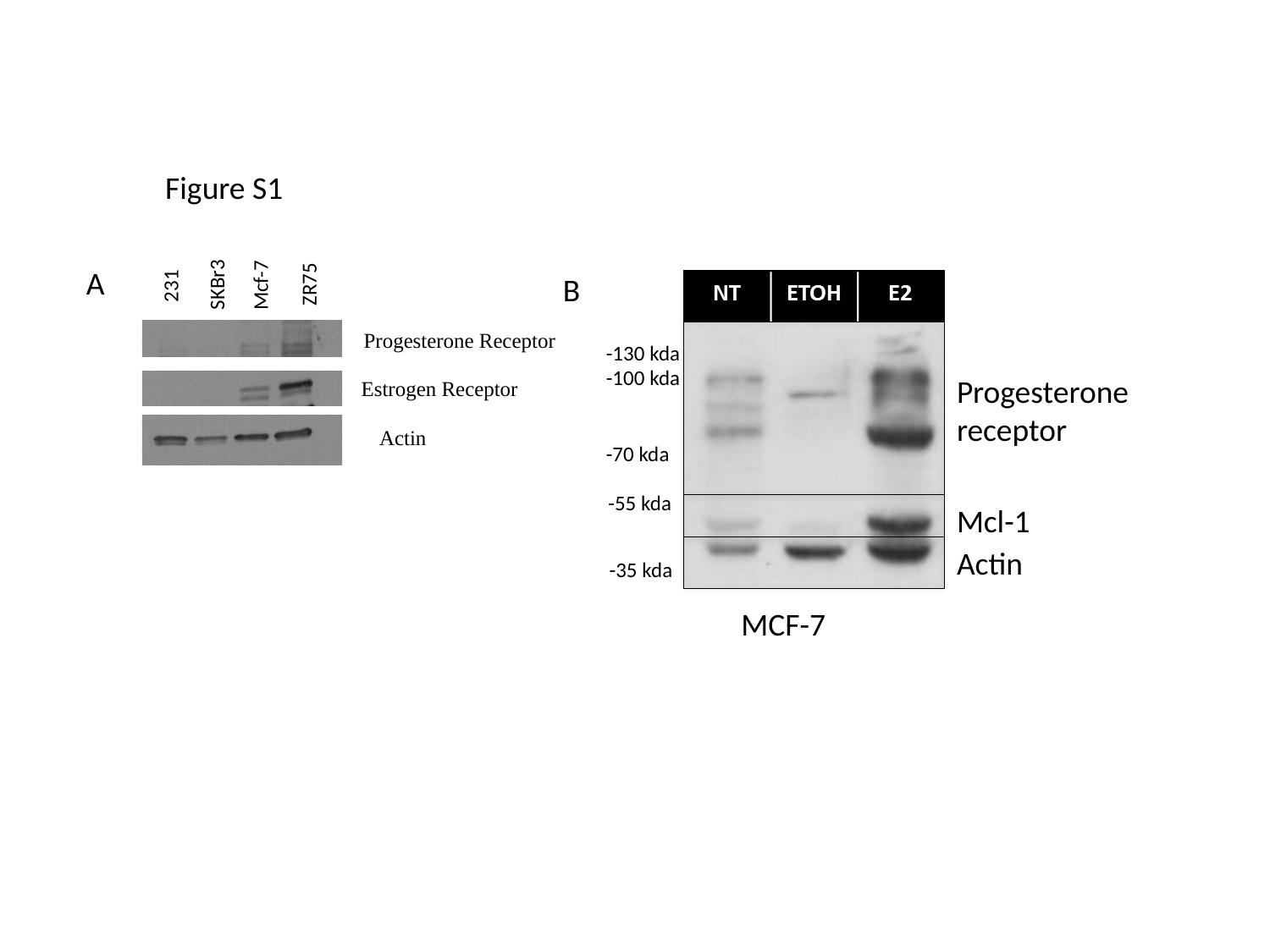

Figure S1
A
B
ZR75
SKBr3
Mcf-7
231
-130 kda
Progesterone Receptor
-100 kda
-70 kda
Progesterone receptor
Estrogen Receptor
Actin
-55 kda
Mcl-1
Actin
-35 kda
MCF-7

## Slide 2
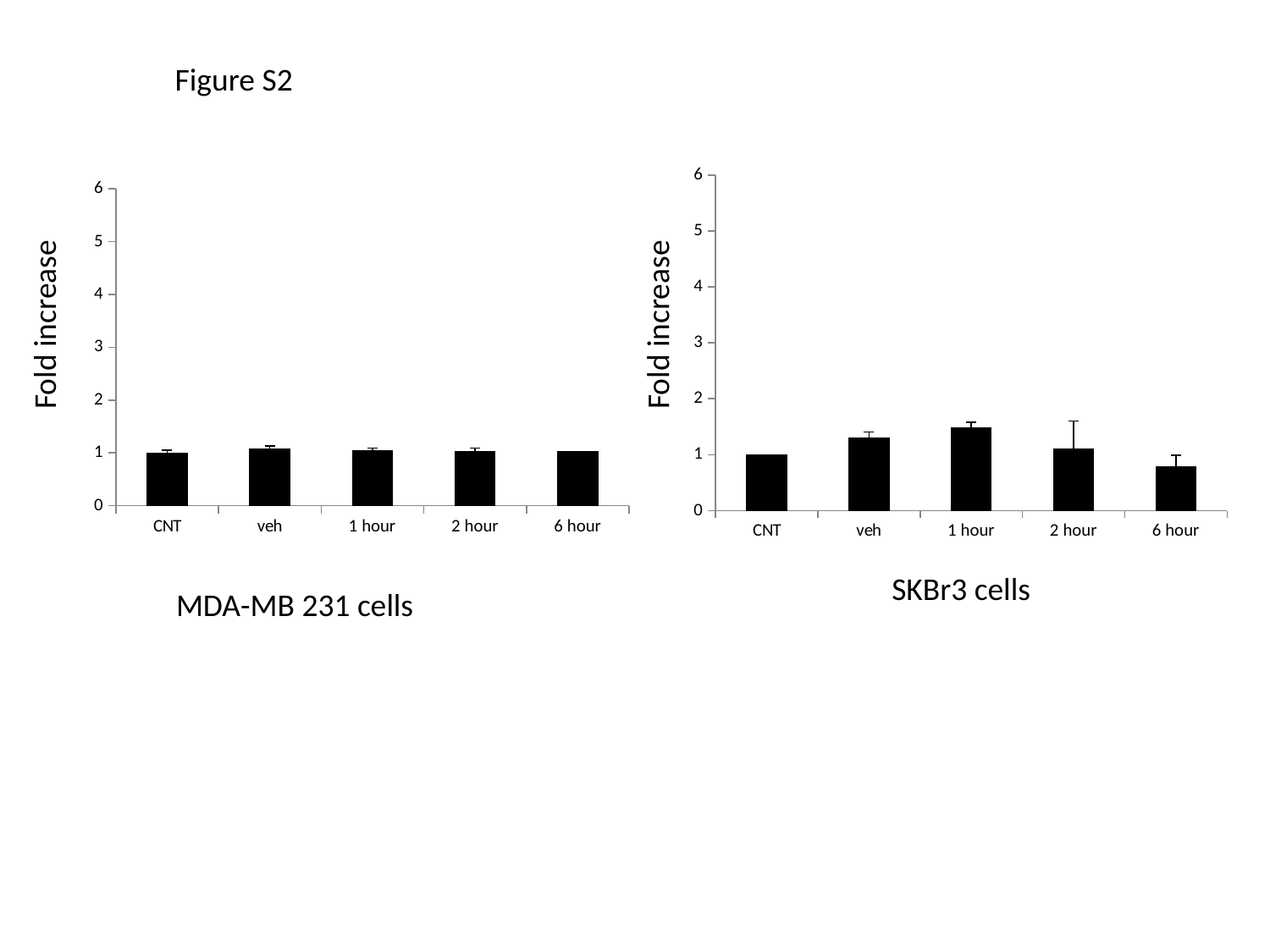

Figure S2
### Chart
| Category | |
|---|---|
| CNT | 1.0 |
| veh | 1.3069761938006152 |
| 1 hour | 1.4835765250211324 |
| 2 hour | 1.1117953152141526 |
| 6 hour | 0.8010711676232711 |
### Chart
| Category | |
|---|---|
| CNT | 1.0 |
| veh | 1.0897075744712466 |
| 1 hour | 1.048717210852486 |
| 2 hour | 1.0352941590409916 |
| 6 hour | 1.0367828707679352 |Fold increase
Fold increase
SKBr3 cells
MDA-MB 231 cells

## Slide 3
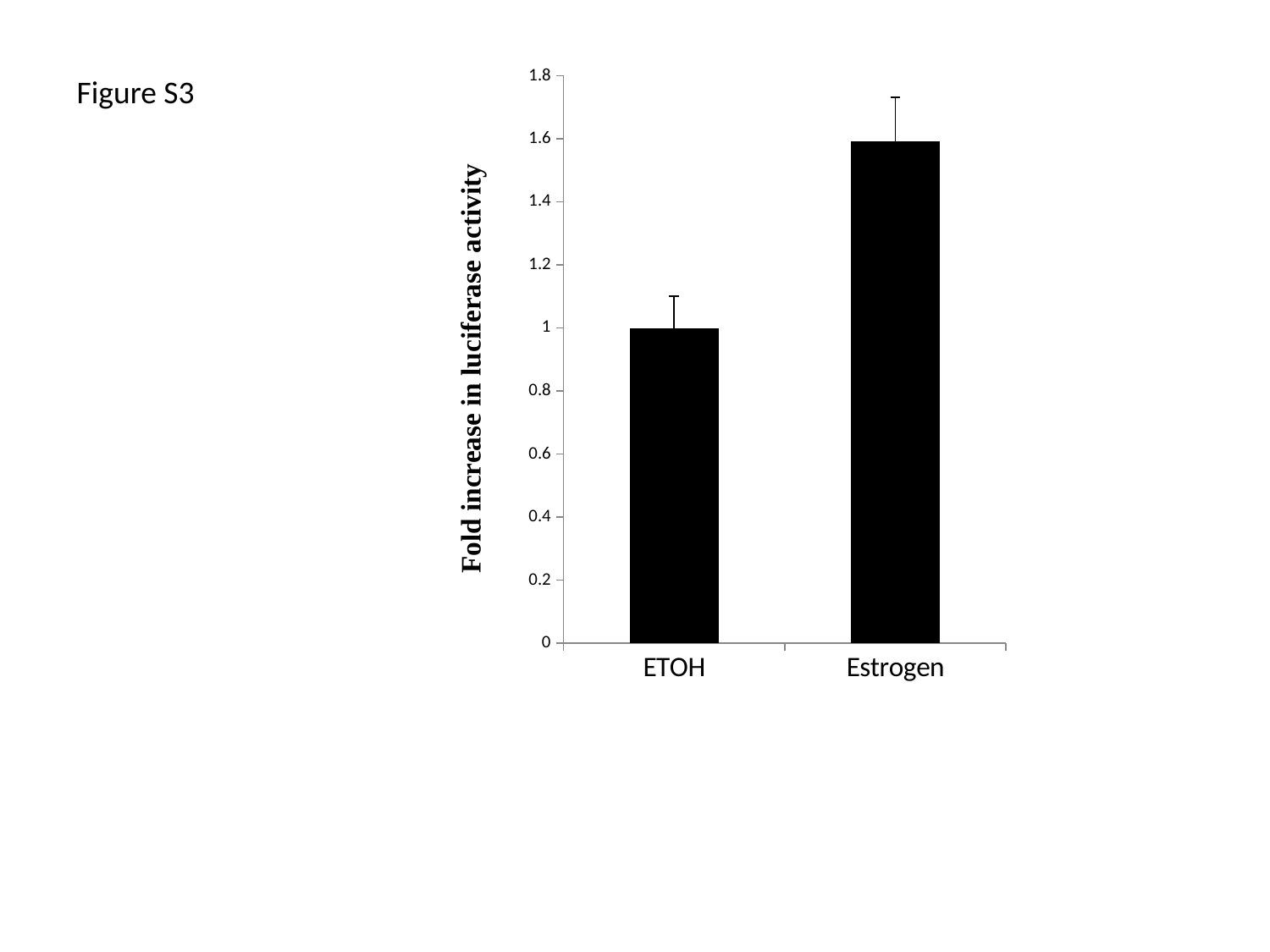

### Chart
| Category | |
|---|---|
| ETOH | 1.0 |
| Estrogen | 1.59135 |Figure S3
Fold increase in luciferase activity

## Slide 4
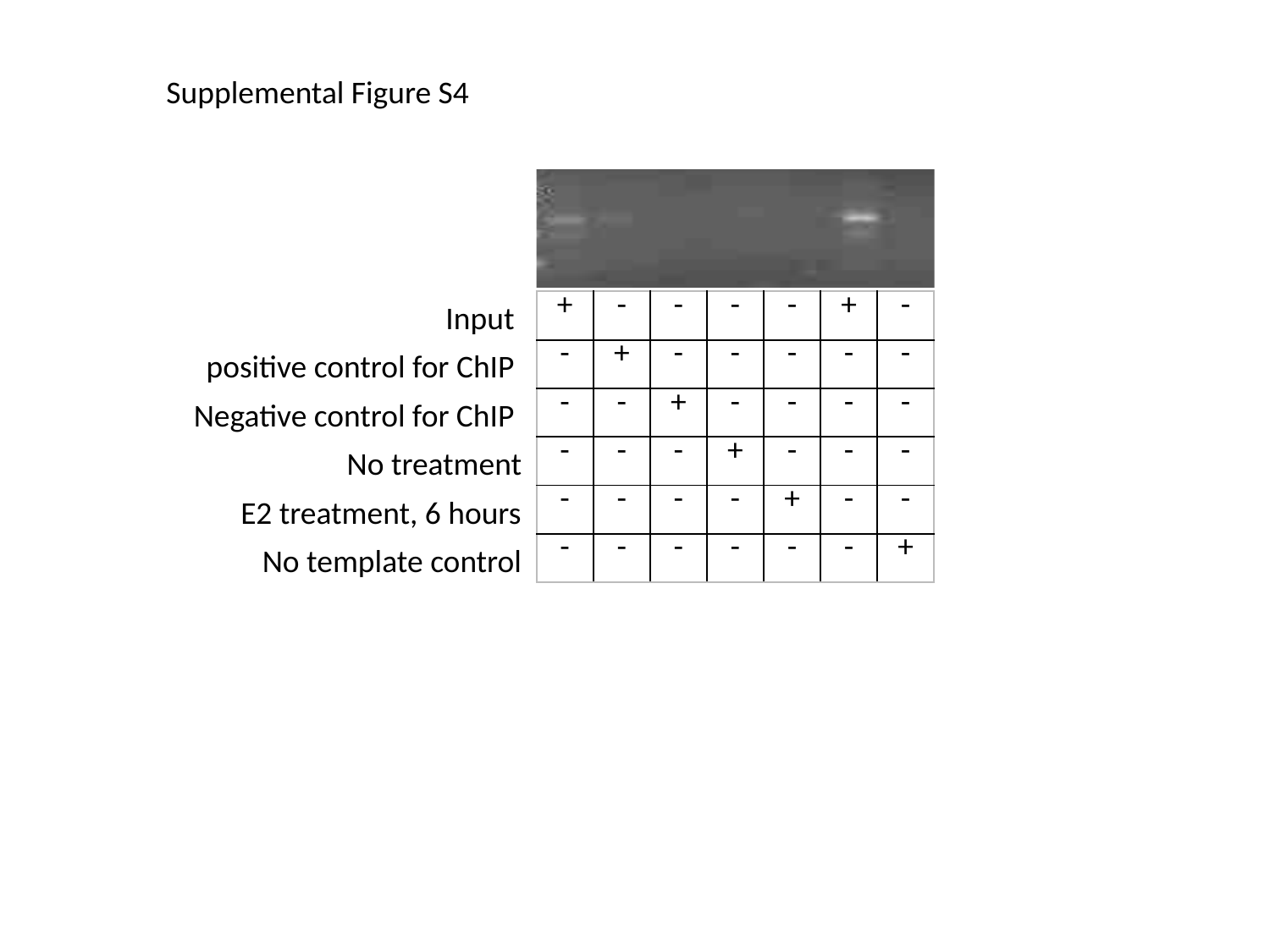

Supplemental Figure S4
Input
positive control for ChIP
Negative control for ChIP
No treatment
E2 treatment, 6 hours
No template control
| + | - | - | - | - | + | - |
| --- | --- | --- | --- | --- | --- | --- |
| - | + | - | - | - | - | - |
| - | - | + | - | - | - | - |
| - | - | - | + | - | - | - |
| - | - | - | - | + | - | - |
| - | - | - | - | - | - | + |

## Slide 5
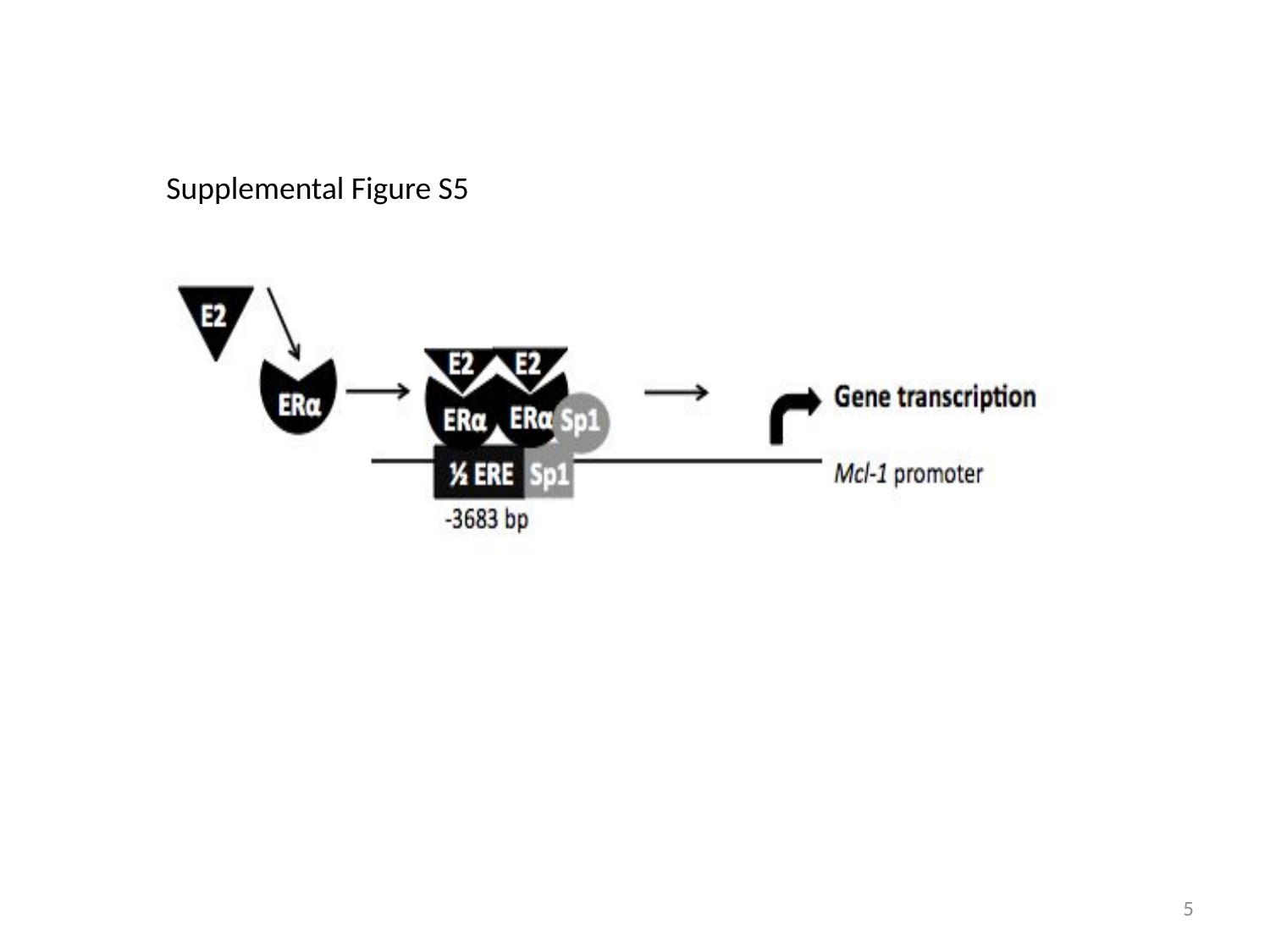

Supplemental Figure S5
5
